# Evaluation of Staining Propensity of Silver Diamine Fluoride With and Without Potassium Iodide in Children (Project Healthy Smiles): Protocol for a Randomized Controlled Trial

**DOI:** 10.2196/51087

**Published:** 2024-07-23

**Authors:** Praveena Navaneethan, Imran Pasha Mohammed, Rekha P Shenoy, Junaid Junaid, Supriya Amanna, Zeyad Alsughier, Shaul Hameed Kolarkodi

**Affiliations:** 1 Department of Public Health Dentistry Yenepoya Dental College Mangaluru India; 2 Department of Orthodontic and Pediatric Dentistry College of Dentistry Qassim University Buraydah Saudi Arabia; 3 Department of Oral and Maxillofacial Diagnostic Sciences College of Dentistry Qassim University Buraydah Saudi Arabia

**Keywords:** silver diamine fluoride, SDF, potassium iodide, KI, tooth discoloration, dental caries, dental esthetics, dental, teeth, healthy smile, staining, treatment, oral health, child

## Abstract

**Background:**

Silver diamine fluoride (SDF) is becoming more widely recognized as a simple, cost-effective approach to minimize sensitivity and arrest caries. However, SDF results in caries that are stained black. Potassium iodide (KI) treatment with SDF may minimize or lessen the staining. However, the effectiveness of KI on staining has not been investigated. Studies demonstrating that potassium iodide reduces the black staining are still insufficient. This paper presents the study protocol for Healthy Smiles, a randomized controlled trial implemented to compare the staining propensity of SDF and SDF+KI.

**Objective:**

This study, Healthy Smiles, aims to evaluate the staining propensity of SDF and SDF+KI using a Nix Mini color sensor among children aged 4 to 6 years. Another objective of the study is to evaluate the caries-arresting effect of SDF and SDF+KI in the treatment of carious primary teeth.

**Methods:**

This study is a randomized controlled trial. A total of 60 children with caries that meet the criteria of the International Caries Detection and Assessment System (code 1 or above) will be randomly assigned to treatment groups, where group 1 will be treated with SDF and group 2 will be treated with SDF+KI. Discoloration of treated lesions will be assessed digitally using a Nix Mini color sensor. Participants will be followed up at 1, 3, and 6 months after treatment to digitally record the ∆L and ∆E values using the Nix Mini color sensor. Data will be analyzed using SPSS (version 28; IBM Corp). Independent sample *t* tests and the Mann-Whitney *U* test will be used to compare the 2 groups.

**Results:**

Enrollment started in October 2023. It is estimated that the enrollment period will be 12 months. Data collection is planned to be completed in 2024.

**Conclusions:**

The presented paper describes Happy Smiles, a project that provides an opportunity to address the aesthetic inconvenience of patients without compromising the effectiveness of the SDF treatment. The trial findings will contribute to the limited evidence base related to discoloration after SDF intervention to improve aesthetic appearances in child oral health. If the results from the trial are promising, it will lead to the development of a model for child oral health and pave the way for further research in child oral health.

**International Registered Report Identifier (IRRID):**

PRR1-10.2196/51087

## Introduction

Developments in our understanding of the pathophysiological processes underlying dental caries led to the development of minimal intervention dentistry, which emphasizes the critical importance of maintaining the integrity of the natural tooth structure and adopts a biological approach in the management of caries lesions [1,2]. Nishino et al [3] described the caries-arresting properties of silver diamine fluoride (SDF) in 1969. SDF is a colorless liquid substance with a pH of 10 that contains 5% to 5.9% fluoride and 24.4% to 28.8% (weight by volume) of silver [4]. The primary reason for SDF’s efficacy in preventing caries is its microbicidal impact on cariogenic biofilm [5,6]. SDF treatment results in a decrease in size and an increase in mineral density and hardness in carious lesions [7].

Children, hospitalized patients, people with mental disorders, people who have received radiation and chemotherapy, and patients with xerostomia, as well as all those with high rates of tooth decay, can benefit from SDF [8]. This is a therapy option in dental clinics and community dental health initiatives due to its low cost and the simplicity of its application. A significant service that dental auxiliaries can be trained to provide is stopping active caries [9]. SDF should be applied every 6 months and no more than 3 weeks apart [10].

Despite the strong evidence for SDF’s antibacterial effectiveness in arresting tooth caries, its clinical application has been relatively limited until now [11]. Location and tooth type have a major impact on parental acceptance of SDF therapy. Parental acceptability of SDF treatment is considerably greater for primary teeth than permanent teeth, and for posterior teeth than anterior teeth in both dentitions [12]. Regardless of the type and location of the teeth, parents of children who had previously exhibited resistant behavior during dental treatment were significantly more receptive of SDF treatment than parents of cooperative children [12].

Contrary to anterior teeth, posterior tooth staining is more widely accepted. Even though anterior tooth discoloration is undesirable, parents prefer SDF treatment to any other complex behavioral approaches like sedation or general anesthesia, which are not appreciated by most parents [13]. The precipitation of silver phosphate is what causes the discoloration that occurs after applying SDF (which has the formula Ag_3_PO_4_) [14].

In order to overcome this problem and increase the clinical use of SDF, a new method of application has been suggested [15]. This includes treating the initial layer of SDF with a layer of potassium iodide (KI), which combines with the free silver ions in SDF and inhibits the precipitation of silver phosphate. Since then, numerous investigations have shown that using KI after SDF creates a yellow silver iodide precipitate and prevents tooth stains from turning dark [10,16]. Following the application of KI in a dose-dependent manner, Detsomboonrat et al [17] noticed a considerable and immediate reduction in staining caused by SDF.

Studies demonstrating that KI reduces black staining are still insufficient. This paper presents the study protocol for Healthy Smiles, a randomized controlled trial implemented to compare the staining propensity of SDF+KI.

The aim of the study is to evaluate the staining propensity of SDF+KI among children aged 4 to 6 years by incorporating artificial intelligence in dentistry as a tool for precise interpretation of the findings, which will add to the evidence pool. The objectives of the study include (1) to evaluate the staining propensity of SDF+KI using a Nix Mini color sensor (Nix Sensor Ltd) among children aged 4 to 6 years, (2) to evaluate the caries-arresting effect of SDF+KI in the treatment of carious primary teeth, and (3) to assess parental satisfaction with dental appearance and color following treatment with SDF versus SDF+KI using a detailed questionnaire (Table 1). We hypothesized that there would be no difference in the staining propensity of SDF+KI among the children.

**Table 1:** Objectives and measures.

Objectives	Measures	Description
1. To evaluate the staining propensity of SDF^a^ and SDF plus KI^b^ using the Nix Mini color sensor among children aged 4 to 6 years.	Child dental examination	At the end of 1, 3, and 6 months after treatment each tooth will be photographed and each tooth’s color change will be measured using the Nix Mini color sensor.
2. To evaluate the caries-arresting effect of SDF and SDF+KI in the treatment of carious primary teeth.	Arrest of carious lesions (6 and 12 months after treatment)	The progression in the size of the carious lesions will be evaluated using the ICDAS^c^ index as stable or progressing. The consistency of the lesions will be evaluated upon gentle probing using a ball-ended World Health Organization probe as soft or hard carious lesions.
3. To assess parental satisfaction with dental appearance and color following treatment with SDF and SDF+KI.	Parental questionnaire using prevalidated 5 point-Likert scale (immediately after treatment)	“You are satisfied with your child's aesthetics after SDF/SDF+KI treatment.” “SDF/SDF+KI application is an easy process.” “SDF/SDF+KI application is pain free for your child.” “SDFSDF+KI taste is acceptable to your child.”

^a^SDF: silver diamine fluoride.

^b^KI: potassium iodide.

^c^ICDAS: International Caries Detection and Assessment System,

## Methods

### Study Design

**Figure 1:**
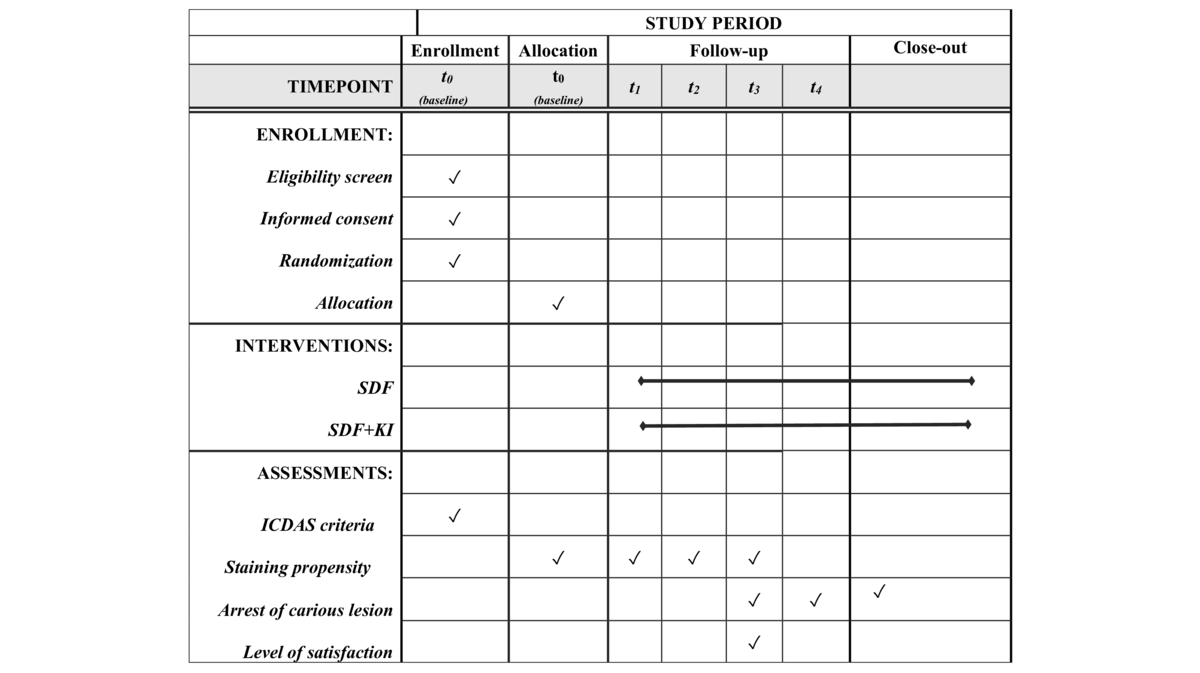
SPIRIT (Standard Protocol Items: Recommendations for Interventional Trials) schedule of enrollment, interventions, and assessments. ICDAS: International Caries Detection and Assessment System; KI: potassium iodide; SDF: silver diamine fluoride; t0: baseline; t1-t4: 1-, 3-, 6-, and 12-month follow-ups.

Healthy Smiles will be carried out for a period of 1 year. The study is an active-controlled, parallel-group randomized controlled trial. A detailed schedule of enrollment, interventions, and assessments is given as a SPIRIT (Standard Protocol Items: Recommendations for Interventional Trials) schedule (Figure 1).

**Figure 2:**
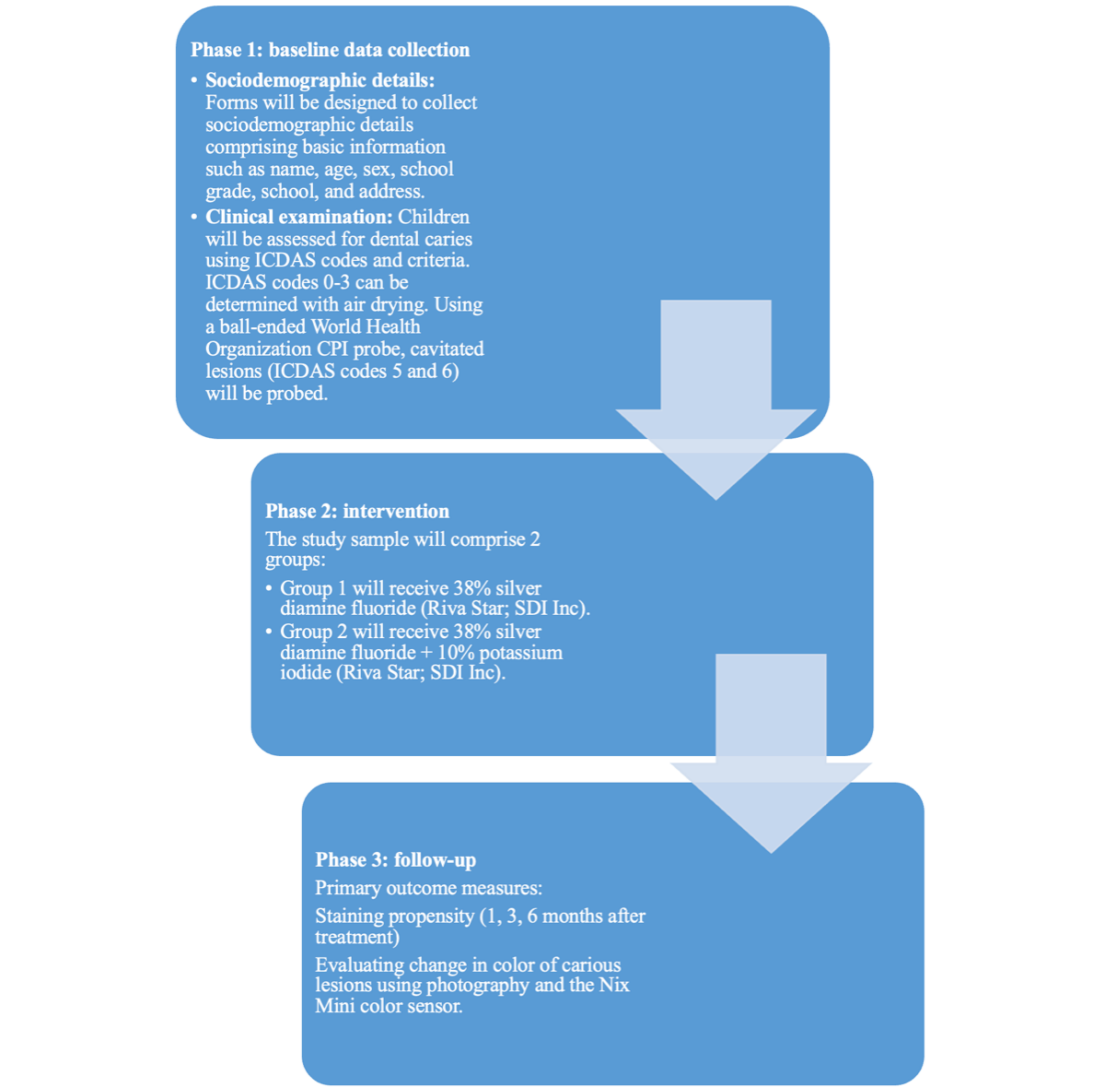
Phases of Healthy Smiles. CPI: Community Periodontal Index; ICDAS: International Caries Detection and Assessment System.

Baseline data will be collected and the children will receive the intervention. The examination will be done by a single examiner with the help of recorders from government-run nonformal preschools and primary schools in Mangaluru, India. This schedule is designed with slight flexibility in order to accommodate any unforeseen lapses or inconveniences. This trial has 3 phases, including data collection, intervention, and follow-up (Figure 2).

### Study Location

The study will be conducted in primary schools and government-run nonformal preschools in Mangaluru, India. Inclusion and exclusion criteria are shown in Textbox 1.

**Inclusion and exclusion criteria.**

**Inclusion criteria**

- Children aged from 4 to 6 years
- Children who meet the criteria for International Caries Detection and Assessment System (ICDAS) code 1 or greater
- Children who have one or more active carious lesions in primary teeth not involving the pulp
- Parents who give consent for participation in the study

**Exclusion criteria**

- Children with preshedding tooth mobility
- Children with signs of pulpal or periapical infection
- Children with gingival or perioral ulceration or stomatitis
- Children who have any history of allergies

### Recruitment: Trial Participants

A total of 120 children will be examined in the government-run nonformal preschools and primary schools by the supervising staff (guide) and the primary investigator. The sample size will be 60 children.

### Randomization

The names of 60 children will be selected randomly with a computer-generated table of random numbers by an independent assistant using the Stat Trek (StatTrek.com) random number generator. The names will be randomly distributed into 2 groups (SDF and SDF+KI) with a lottery method for the next phase. For the trial, 2 groups will be assigned: group 1 and group 2. The assistant, who is independent of the trial, will be responsible for giving each child a serial number indicating his or her allocation. Blinding of the examiner or the children will be impossible during the intervention and follow-up due to the different natures of the materials. However, the assistant, who will be responsible for giving and collecting the questionnaires from the parents or guardians, as well as the statistician, will not know the group to which the child belonged.

### Standardization and Calibration

Clinical examinations using the ICDAS codes and criteria will be performed among children with active dental caries by the primary investigator and the supervising staff (guide). Standardization and calibration for the ICDAS index will be done before commencing the study. Calibration will be done at the Department of Public Health Dentistry. For this, 10 participants will be chosen in the 4- to 6-year age group with dental caries with an ICDAS code of 1 or greater, for whom the examiners will record the index. For determination of intra-examiner variability, these participants will be recalled on different days and the same examiner will repeat the examination. Interexaminer reliability will be assessed and compared with the gold standard examiner (guide). The κ coefficient value will be calculated, and a κ coefficient of 0.80 and above will be accepted as a reliable level of agreement.

### Statistical Power

The sample size was calculated with G*Power (version 3; Universität Düsseldorf). Based on the results of Aly and Yousry [18], at a 5% level of significance and 95% power with an effect size of 1.594, (calculated by considering that the mean lightness value of one group was 71.3, SD 14.2 and the other group was 53.6, SD 6.7), and the primary end point being an evaluation of the staining propensity of SDF+KI at the end of 1, 3, and 6 months after treatment, with a 95% chance of detecting an increase in the mean lightness value from 53.6 in the control group to 71.3 in the experimental group, the minimum sample size in each group is 16. The expected dropout rate is 10%, so after considering the dropout rate, the sample size in each group is 30. Thus, the total sample required for the intervention will be 60 children (N=60).

For the secondary objective, with a significance level of 5%, a power of 80%, and an estimated critical *Z* value of 3.193, the study required 48 teeth, or 24 teeth per group. Finally, assuming 40% loss to follow-up, a sample size of 34 teeth was gathered in each group [19] to evaluate the caries-arresting effect of SDF+KI in the treatment of carious primary teeth at 6 and 12 months after treatment.

### Data Collection

**Figure 3:**
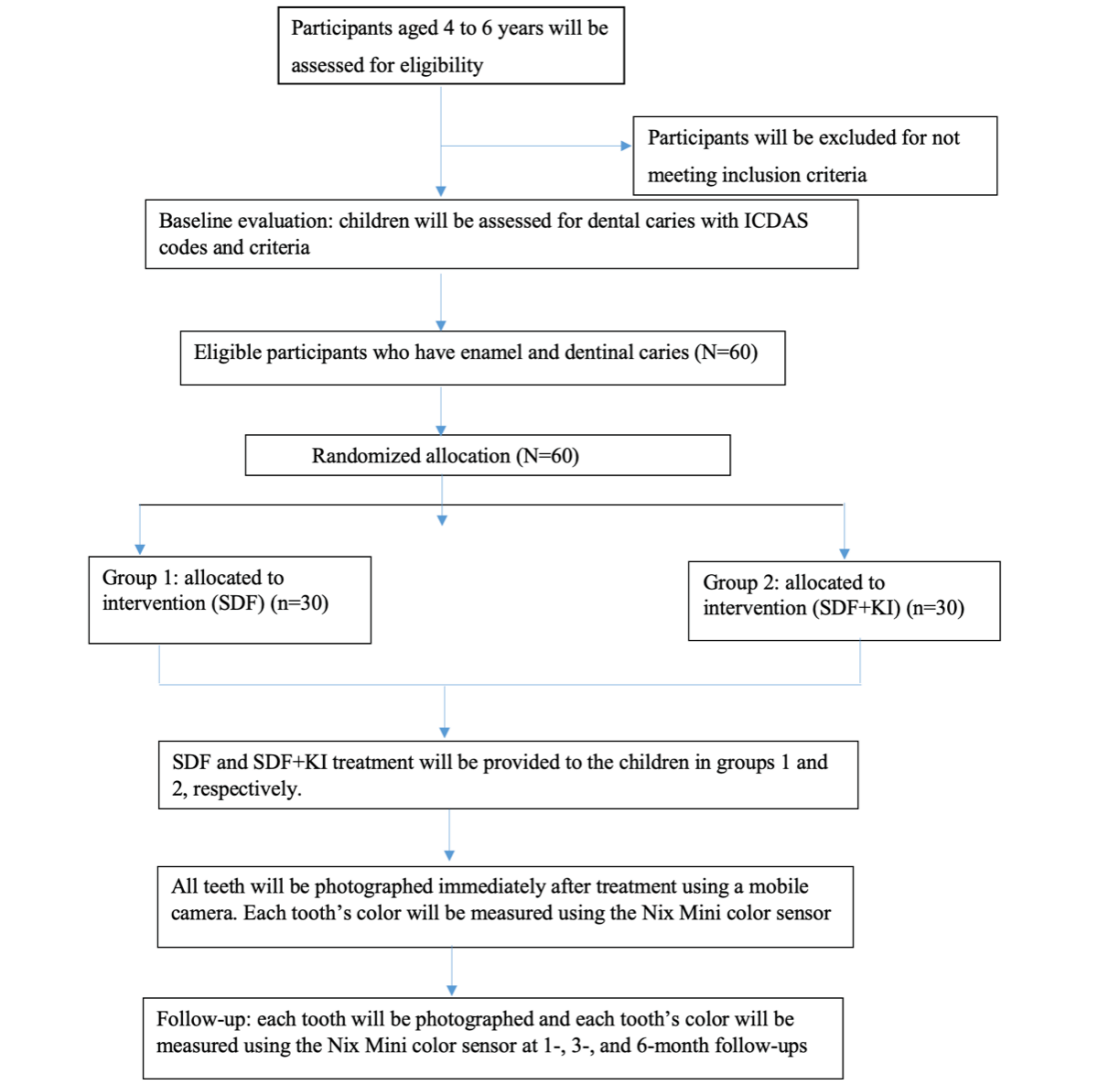
CONSORT (Consolidated Standards of Reporting Trials) flowchart of Healthy Smiles. ICDAS: International Caries Detection and Assessment System; KI: potassium iodide; SDF: silver diamine fluoride.

Data were collected with research data management software, including REDCap (Research Electronic Data Capture), a web-based application designed specifically for clinical research for consent tracking and data validation. A detailed schedule of data collection and a CONSORT (Consolidated Standards of Reporting Trials) flowchart of the study protocol, including baseline data collection, intervention, and follow-up, is shown in Figure 3.

### Phase 1: Baseline Data Collection

#### Sociodemographic Details

The details about the Healthy Smiles will be clearly explained at a parent-teacher association meeting. Parents who give consent for participation will be considered. A standard form will be designed on Google Forms to collect sociodemographic details, comprising basic information such as name, age, sex, school grade, school, and address. Data will be protected and kept confidential.

#### Clinical Examinations

Clinical examinations will be performed by visual inspection under visible light, with the children in an upright position. The children will be assessed for dental caries using ICDAS codes and criteria. ICDAS codes 0-3 can be determined with air drying. Using a ball-ended World Health Organization (WHO) Community Periodontal Index probe, cavitated lesions (ICDAS codes 5 and 6) will be probed.

### Phase 2: Intervention

The Healthy Smiles study sample will comprise 2 groups: group 1 will receive 38% SDF (Riva Star; SDI Inc); group 2 will receive 38% SDF with 10% KI (Riva Star; SDI Inc).

#### Method of Application

In group 1, we will use cotton gauze to carefully clean and dry the affected tooth surface. The gingival tissue of the tooth will be protected with petroleum jelly. An applicator will be dipped into the SDF and 2-3 mg will be applied to the lesion with a microbrush, which will be used to paint the solution over the carious lesion; then, we will wait for 1 minute for absorption.

In group 2, we will apply SDF with the same technique. After the SDF application, KI will be applied using a separate microbrush, which will be saturated with the solution and will be used to paint the solution over the carious lesion. No rinse will be performed and special instructions will be given following application.

After treatment, an individual report on the child’s oral health will be provided, and after-treatment instructions will be given to the parents. If there is any adverse drug reaction associated with the SDF treatment, the parents will be asked to contact the principal investigator immediately by telephone.

All teeth will be photographed immediately after treatment. A high resolution mobile camera will be used to capture all of the images throughout the study. The high-resolution mobile camera features a 64-megapixel primary camera with an 8-megapixel secondary depth camera and an 8× optical zoom to provide images at a 2340×1080-pixel resolution.

Each tooth’s color will be measured using a Nix Mini color sensor. The Nix Mini color sensor is a wireless colorimeter that uses artificial intelligence to measure the exact color of surfaces and transmits the digital color values to a smartphone or tablet using Bluetooth.

Measurements will be calibrated in accordance with the manufacturer’s instructions to determine the first shade of the carious lesion. The ∆L and ∆E values will be measured 3 times by a single operator, and the average values will be noted. Despite the fact that SDF treatment had no adverse effects in other studies, if any adverse events occur, the primary investigator will duly report them to the institutional ethics committee.

### Phase 3: Follow-Up

#### Primary Outcome Measures

Staining propensity will be measured 1, 3, and 6 months after treatment. Evaluation of the change in color of the carious lesions will use photographs and the Nix Mini color sensor.

#### Secondary Outcome Measures

The arrest of carious lesions will be measured 6 and 12 months after treatment. The size of the carious lesions will be evaluated using the ICDAS index as stable or progressing. The consistency of the lesions will be evaluated with gentle probing using a ball-ended WHO probe as soft or hard. The level of satisfaction will be measured immediately after intervention. Parental satisfaction with dental appearance and color following treatment with SDF and SDF+KI will be assessed using a prevalidated 4 item, 5-point Likert-scale questionnaire in English developed by Clemens et al [20] in 2018.

### Data Analysis

Data will be analyzed using SPSS (version 28; IBM Corp). Descriptive statistics will include the mean and SD for continuous data and frequency and percentage for categorical data. An independent-sample 1-tailed *t* test or the Mann-Whitney *U* test will be used for comparing the 2 groups. *P* values will be considered statistically significant at ≤.05.

### Ethical Considerations

Ethical approval of this trial was obtained from the institutional ethics committee (REF/2022/06/05559) of the Yenepoya (Deemed To Be University). Parent or guardians will be asked to provide a written informed consent form, which also covers the secondary analysis. All procedures will be conducted in accordance with the ethical standards of the Declaration of Helsinki and in accordance with relevant guidelines and regulations.

A participant information sheet describing the purpose of the study; details of the research, including the secondary analysis; procedures; and benefits and adverse effects, specifically tooth discoloration, will be given to the parents or legal guardians of the children. Their concerns about the adverse effects will be answered. Informed consent will be sought before the trial’s start. The study’s participants, including the children and their legal guardians, are free to discontinue at any moment during the study. Additionally, they are free to seek out extra dental treatment in accordance with their own preferences.

The drugs will be obtained by the primary investigator and are not sponsored. Drugs are stored at room temperature according to the manufacturer’s instructions. The manufacturer played no role in this study. The data safety sheet from the manufacturer for the SDF is provided in Multimedia Appendix 1. The drug controller general of India does not prohibit SDF use. Furthermore, no dental professional organizations or research associations have yet issued any SDF recommendations.

The privacy of the children will be respected. The data collected from the children will be kept confidential and will be shared only among the members of the research team, ethics committee, and regulatory authorities. No one else shall be privy to the children’s details. The photographs will be masked.

There will be a clinical evaluation for dental caries, during which the principal investigator will administer the necessary treatment if any pulpal involvement or abscess is found. The right to refuse or right to withdraw at any point of time will be given to the children. No monetary compensation will be provided in case of withdrawal. Participants who stay in the study will receive no monetary compensation, but will receive referral cards for further dental treatments at our institution free of cost.

### Risks and Discomforts

There are no major known risks to the study. There are minor risks, such as black stains on the carious tooth surface or temporary stains on the skin. Temporary stains on the skin will disappear within 48 hours without treatment. Participants who are allergic to the products will be excluded from the study.

## Results

Enrollment started in October 2023. It is estimated that the enrollment period will be 12 months. Data collection is planned to be completed in 2024.

## Discussion

### Principal Findings

Riva Star is a commercially available product that contains 38% SDF, so it will be used in the control group. Riva Star is the only commercially available product with 38% SDF+KI. As a result, it will be used in the case group. With oral administration, the average lethal dose (LD50) is 520 mg/kg. One drop (25 μL) contains 9.5 mg SDF, which is sufficient to treat 5 teeth. Considering that the smallest child with caries weighs around 10 kg, the dose would be 0.95 mg/kg. Hence, the relative safety margin for administering a complete drop to a 10-kg child is 380 mg/kg, so for an LD50 of 0.95 mg/kg the dosage is 400 times the safety margin. The actual dose is likely to be significantly lower. The recommended limit is 1 drop (25 μL) for 10 kg of body weight at every treatment visit. The maximum applied dose for 3 teeth is 2.37 mg, which would allow for over 400 applications [21].

Silver ions in the SDF solution have the potential to darken the tooth structure. It has been proposed that the KI solution can react with SDF to generate a bright yellow solid product (silver iodide), which could reduce the excess free silver ions that cause the black staining. Despite the presence of brilliant yellow precipitates following KI application, staining of tooth surfaces was still observed in an SDF+KI treatment group in a previous investigation. While KI is anticipated to eliminate SDF staining, its effect has not previously been quantified [22].

Patients are concerned about the aesthetic appearance of a restoration. Rather than analyzing color variations with the naked eye, which is typically subjective, we will quantify color changes using smart device measurements incorporating artificial intelligence, as these are more precise and repeatable [23].The Nix Mini color sensor is a wireless colorimeter that uses artificial intelligence to measure the exact color of surfaces and communicates the digital color values through Bluetooth to a smartphone or tablet. The color sensor blocks out ambient light and provides its own calibrated light source using the industry-standard 45/0° measurement. The device provides color readouts in International Commission on Illumination L*a*b* (CIELAB) format and calculates color differences in ∆E2000 [10]. The Nix Mini color sensor will be used immediately following treatment. From the Nix Mini color data, ∆L and ∆E values will be recorded and compared among the groups.

This is the first randomized controlled trial in which artificial intelligence–based sensing equipment and qualitative opinions of both the dentist (investigator) and parents will be ascertained. This study will help dentists and parents make the important clinical decision to choose a 2-step, noninvasive, low-cost treatment that is more acceptable and practicable in places lacking complete dental facilities, for hospital patients, and for low-income children with restricted access to comprehensive dental care.

### Strengths and Limitations of the Study

First, this is a randomized controlled trial that quantifies the color change after SDF and SDF+KI treatment to help maximize the acceptability of the preventive treatment program.

Second, the use of ICDAS codes as a measurement of the stability of the lesion size as a standard for arrested caries is another feature of the study design. Prior studies only considered hardness and color, which are challenging to calibrate. The calibrated examiners in this investigation were able to provide an accurate evaluation of lesion size stability.

Finally, this study will use the Nix Mini color sensor, which senses colors and readouts in CIELAB; ∆L and ∆E values will be quantitatively recorded.

A limitation of the study is that it is a short-term trial; longitudinal studies will have to be done to report the long-term effect on discoloration of SDF.

### Conclusions

Healthy Smiles provides an opportunity to address the aesthetic inconvenience of patients without compromising the effectiveness of SDF treatment. This trial’s findings will contribute to the limited evidence base on discoloration after SDF intervention to improve aesthetic appearance in child oral health. If the results from the trial are promising, it will lead to the development of a model for child oral health and pave the way for further research in child oral health.

## Appendix

Multimedia Appendix 1 Certificate of analysis for quality control of silver diamine fluoride.

## Abbreviations

CIELAB: International Commission on Illumination L*a*b*
CONSORT: Consolidated Standards of Reporting Trials
ICDAS: International Caries Detection and Assessment System
KI: potassium iodide
REDCap: Research Electronic Data Capture
SDF: silver diamine fluoride
SPIRIT: Standard Protocol Items: Recommendations for Interventional Trials
WHO: World Health Organization
