# Supplementary material for: Evaluation of Staining Propensity of Silver Diamine Fluoride With and Without Potassium Iodide in Children (Project Healthy Smiles): Protocol for a Randomized Controlled Trial
Source: JMIR Res Protoc. 2024 Jul 23;13:e51087. doi: 10.2196/51087 (PMC11303896; doi:10.2196/51087)
Supplement: Multimedia Appendix 1 [file resprot_v13i1e51087_app1.pdf]

Riva Star
SDI Limited

Version No: **7.1.1.1**
Safety Data Sheet (Conforms to Regulation (EU) No 2015/830)

Issue Date: **03/09/2020**
Print Date: **02/10/2020**
L.REACH.GBR.EN

SECTION 1 Identification of the substance / mixture and of the company / undertaking

1.1. Product Identifier

**Product name**	Riva Star
**Synonyms**	Not Available
**Proper shipping name**	AMMONIA SOLUTION, relative density between 0.880 and 0.957 at 15 °C in water, with more than 10% but not more than 35% ammonia
**Other means of identification**	Not Available

1.2. Relevant identified uses of the substance or mixture and uses advised against

**Relevant identified uses**	(Riva Star is comprised of Riva Star Step 1 and Riva Star Step 2) for use as a tooth desensitising agent.
**Uses advised against**	Not Applicable

1.3. Details of the supplier of the safety data sheet

**Registered company name**	**SDI Limited**	**SDI (North America) Inc.**	**SDi**
**Address**	3-15 Brunsdon Street Bayswater VIC 3153 Australia	1279 Hamilton Parkway Itasca IL 60143 United States	Rua Dr. Virgílio de Carvalho Pinto, 612 Pinheiros, Sao Paulo 05415-020 Brazil
**Telephone**	+61 3 8727 7111 (Business Hours)	+1 630 361 9200 (Business hours)|1 800 228 5166	+55 11 3092 7100 (Business Hours)
**Fax**	+61 3 8727 7222	+1 630 361 9222	+55 11 3092 7101
**Website**	www.sdi.com.au	http://www.sdi.com.au	http://www.sdi.com.au/
**Email**	info@sdi.com.au	USA.Canada@sdi.com.au	Brasil@sdi.com.au

**Registered company name**	**SDI Dental Limited**
**Address**	Block 8, St Johns Court Santry Dublin 9 Ireland
**Telephone**	+353 1 886 9577 (Business Hours)|800 0225 5734
**Fax**	Not Available
**Website**	http://www.sdi.com.au/
**Email**	Ireland@sdi.com.au

1.4. Emergency telephone number

**Association / Organisation**	**SDI Limited**	**SDi**	**SDI Dental Limited**
**Emergency telephone numbers**	+61 3 8727 7111	+61 3 8727 7111	+61 3 8727 7111
**Other emergency telephone numbers**	ray.cahill@sdi.com.au	Not Available	Not Available

SECTION 2 Hazards identification

2.1. Classification of the substance or mixture

**Classification according to regulation (EC) No 1272/2008 [CLP] and amendments [1]**	H290 - Metal Corrosion Category 1, H301 - Acute Toxicity (Oral) Category 3, H314 - Skin Corrosion/Irritation Category 1B, H400 - Acute Aquatic Hazard Category 1
Legend:	*1. Classification by vendor; 2. Classification drawn from Regulation (EU) No 1272/2008 - Annex VI*

2.2. Label elements

**Hazard pictogram(s)**	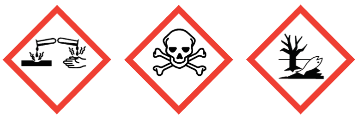
**Signal word**	**Danger**

Hazard statement(s)

**H290**	May be corrosive to metals.
**H301**	Toxic if swallowed.
**H314**	Causes severe skin burns and eye damage.
**H400**	Very toxic to aquatic life.

Supplementary statement(s)

Not Applicable

Precautionary statement(s) Prevention

**P260**	Do not breathe mist/vapours/spray.
**P270**	Do not eat, drink or smoke when using this product.
**P280**	Wear protective gloves/protective clothing/eye protection/face protection.
**P234**	Keep only in original packaging.
**P273**	Avoid release to the environment.

Precautionary statement(s) Response

**P301+P310**	IF SWALLOWED: Immediately call a POISON CENTER/doctor/physician/first aider.
**P301+P330+P331**	IF SWALLOWED: Rinse mouth. Do NOT induce vomiting.
**P303+P361+P353**	IF ON SKIN (or hair): Take off immediately all contaminated clothing. Rinse skin with water [or shower].
**P305+P351+P338**	IF IN EYES: Rinse cautiously with water for several minutes. Remove contact lenses, if present and easy to do. Continue rinsing.
**P321**	Specific treatment (see advice on this label).
**P363**	Wash contaminated clothing before reuse.
**P390**	Absorb spillage to prevent material damage.
**P391**	Collect spillage.
**P304+P340**	IF INHALED: Remove person to fresh air and keep comfortable for breathing.

Precautionary statement(s) Storage

**P405**	Store locked up.

Precautionary statement(s) Disposal

**P501**	Dispose of contents/container to authorised hazardous or special waste collection point in accordance with any local regulation.

2.3. Other hazards

Ingestion may produce health damage*.

Cumulative effects may result following exposure*.

REACh - Art.57-59: The mixture does not contain Substances of Very High Concern (SVHC) at the SDS print date.

SECTION 3 Composition / information on ingredients

3.1.Substances

See 'Composition on ingredients' in Section 3.2

3.2.Mixtures

**1.CAS No**<br>**2.EC No**<br>**3.Index No**<br>**4.REACH No**	**%[weight]**	**Name**	**Classification according to regulation (EC) No 1272/2008 [CLP] and amendments**
Not Available		each 0.05ml capsule of Riva Star Step 1 contains:	Not Applicable
1.7775-41-9<br>2.231-895-8<br>3.Not Available<br>4.Not Available	35-40	silver(I) fluoride *****	Acute Toxicity (Inhalation) Category 3, Metal Corrosion Category 1, Serious Eye Damage Category 1, Skin Corrosion/Irritation Category 1A, Acute Toxicity (Oral) Category 3, Acute Toxicity (Dermal) Category 3; H331, H290, H318, H314, H301, H311 [1]
1.1336-21-6<br>2.215-647-6<br>3.007-001-01-2<br>4.01-2119982985-14-XXXX	15-20	ammonia	Metal Corrosion Category 1, Serious Eye Damage Category 1, Acute Aquatic Hazard Category 1, Skin Corrosion/Irritation Category 1B, Acute Toxicity (Inhalation) Category 4; H290, H318, H400, H314, H332 [1]
1.7732-18-5<br>2.231-791-2<br>3.Not Available<br>4.Not Available	balance	water	Not Applicable
Not Available		Riva Star Step 2 contains:	Not Applicable
Not Available	100	Ingredients determined not to be hazardous	Not Applicable
Legend:	*1. Classification by vendor; 2. Classification drawn from Regulation (EU) No 1272/2008 - Annex VI; 3. Classification drawn from C&L; * EU*		

Continued...

	IOELVs available

SECTION 4 First aid measures

4.1. Description of first aid measures

**Eye Contact**	If this product comes in contact with the eyes:<br>▸ Immediately hold eyelids apart and flush the eye continuously with running water.<br>▸ Ensure complete irrigation of the eye by keeping eyelids apart and away from eye and moving the eyelids by occasionally lifting the upper and lower lids.<br>▸ Continue flushing until advised to stop by the Poisons Information Centre or a doctor, or for at least 15 minutes.<br>▸ Transport to hospital or doctor without delay.<br>▸ Removal of contact lenses after an eye injury should only be undertaken by skilled personnel.
**Skin Contact**	If skin contact occurs:<br>▸ Immediately remove all contaminated clothing, including footwear.<br>▸ Flush skin and hair with running water (and soap if available).<br>▸ Seek medical attention in event of irritation.
**Inhalation**	▸ If fumes or combustion products are inhaled remove from contaminated area.<br>▸ Seek medical attention.
**Ingestion**	▸ If swallowed do **NOT** induce vomiting.<br>▸ If vomiting occurs, lean patient forward or place on left side (head-down position, if possible) to maintain open airway and prevent aspiration.<br>▸ Observe the patient carefully.<br>▸ Never give liquid to a person showing signs of being sleepy or with reduced awareness; i.e. becoming unconscious.<br>▸ Give water to rinse out mouth, then provide liquid slowly and as much as casualty can comfortably drink.<br>▸ Seek medical advice.

4.2 Most important symptoms and effects, both acute and delayed

See Section 11

4.3. Indication of any immediate medical attention and special treatment needed

Treat symptomatically.

SECTION 5 Firefighting measures

5.1. Extinguishing media

- Dry chemical powder.
- BCF (where regulations permit).
- Carbon dioxide.

5.2. Special hazards arising from the substrate or mixture

**Fire Incompatibility**	None known.

5.3. Advice for firefighters

**Fire Fighting**	▸ Alert Fire Brigade and tell them location and nature of hazard.<br>▸ Wear full body protective clothing with breathing apparatus.<br>▸ Prevent, by any means available, spillage from entering drains or water course.<br>▸ Use fire fighting procedures suitable for surrounding area.<br>▸ **Do not** approach containers suspected to be hot.<br>▸ Cool fire exposed containers with water spray from a protected location.<br>▸ If safe to do so, remove containers from path of fire.<br>▸ Equipment should be thoroughly decontaminated after use.
**Fire/Explosion Hazard**	▸ Non combustible.<br>▸ Not considered to be a significant fire risk.<br>▸ Acids may react with metals to produce hydrogen, a highly flammable and explosive gas.<br>▸ Heating may cause expansion or decomposition leading to violent rupture of containers.<br>▸ May emit corrosive, poisonous fumes. May emit acrid smoke.<br>Decomposition may produce toxic fumes of:<br>nitrogen oxides (NOx)<br>ammonia

SECTION 6 Accidental release measures

6.1. Personal precautions, protective equipment and emergency procedures

See section 8

6.2. Environmental precautions

See section 12

6.3. Methods and material for containment and cleaning up

**Minor Spills**	▸ Drains for storage or use areas should have retention basins for pH adjustments and dilution of spills before discharge or disposal of material.<br>▸ Check regularly for spills and leaks.<br>▸ Clean up all spills immediately.<br>▸ Avoid breathing vapours and contact with skin and eyes.<br>▸ Control personal contact with the substance, by using protective equipment.<br>▸ Contain and absorb spill with sand, earth, inert material or vermiculite.

Continued...

	- Wipe up.<br>- Place in a suitable, labelled container for waste disposal.
**Major Spills**	- Clear area of personnel and move upwind.<br>- Alert Fire Brigade and tell them location and nature of hazard.<br>- Wear full body protective clothing with breathing apparatus.<br>- Prevent, by any means available, spillage from entering drains or water course.<br>- Stop leak if safe to do so.<br>- Contain spill with sand, earth or vermiculite.<br>- Collect recoverable product into labelled containers for recycling.<br>- Neutralise/decontaminate residue (see Section 13 for specific agent).<br>- Collect solid residues and seal in labelled drums for disposal.<br>- Wash area and prevent runoff into drains.<br>- After clean up operations, decontaminate and launder all protective clothing and equipment before storing and re-using.<br>- If contamination of drains or waterways occurs, advise emergency services.

6.4. Reference to other sections

Personal Protective Equipment advice is contained in Section 8 of the SDS.

SECTION 7 Handling and storage

7.1. Precautions for safe handling

**Safe handling**	- Avoid all personal contact, including inhalation.<br>- Wear protective clothing when risk of exposure occurs.<br>- Use in a well-ventilated area.<br>- Avoid contact with moisture.<br>- Avoid contact with incompatible materials.<br>- When handling, **DO NOT** eat, drink or smoke.<br>- Keep containers securely sealed when not in use.<br>- Avoid physical damage to containers.<br>- Always wash hands with soap and water after handling.<br>- Work clothes should be laundered separately. Launder contaminated clothing before re-use.<br>- Use good occupational work practice.<br>- Observe manufacturer's storage and handling recommendations contained within this SDS.<br>- Atmosphere should be regularly checked against established exposure standards to ensure safe working conditions are maintained.
**Fire and explosion protection**	See section 5
**Other information**	**Do not** store in direct sunlight.<br>Store in a dry and well ventilated-area, away from heat and sunlight.

7.2. Conditions for safe storage, including any incompatibilities

**Suitable container**	- **DO NOT** repack. Use containers supplied by manufacturer only.
**Storage incompatibility**	- Avoid contact with copper, aluminium and their alloys.

7.3. Specific end use(s)

See section 1.2

SECTION 8 Exposure controls / personal protection

8.1. Control parameters

Ingredient	DNELs<br>Exposure Pattern Worker	PNECs<br>Compartment
Not Available	Not Available	Not Available

* Values for General Population

Occupational Exposure Limits (OEL)

INGREDIENT DATA

Source	Ingredient	Material name	TWA	STEL	Peak	Notes
UK Workplace Exposure Limits (WELs)	silver(I) fluoride	Fluoride (inorganic as F)	2.5 mg/m3	Not Available	Not Available	Not Available
UK Workplace Exposure Limits (WELs)	silver(I) fluoride	Silver (soluble compounds as Ag)	0.01 mg/m3	Not Available	Not Available	Not Available
EU Consolidated List of Indicative Occupational Exposure Limit Values (IOELVs)	silver(I) fluoride	Inorganic Fluorides	2.5 mg/m3	Not Available	Not Available	Skin
EU Consolidated List of Indicative Occupational Exposure Limit Values (IOELVs)	silver(I) fluoride	Silver (soluble compounds as Ag)	0.01 mg/m3	Not Available	Not Available	Not Available

Emergency Limits

Ingredient	Material name	TEEL-1	TEEL-2	TEEL-3
ammonia	Ammonium hydroxide	61 ppm	330 ppm	2,300 ppm

Ingredient	Original IDLH	Revised IDLH
silver(I) fluoride	10 mg/m3	Not Available

Continued...

Ingredient	Original IDLH	Revised IDLH
ammonia	Not Available	Not Available
water	Not Available	Not Available

Occupational Exposure Banding

Ingredient	Occupational Exposure Band Rating	Occupational Exposure Band Limit
ammonia	E	≤ 0.1 ppm
Notes:	*Occupational exposure banding is a process of assigning chemicals into specific categories or bands based on a chemical's potency and the adverse health outcomes associated with exposure. The output of this process is an occupational exposure band (OEB), which corresponds to a range of exposure concentrations that are expected to protect worker health.*	

MATERIAL DATA

8.2. Exposure controls

8.2.1. Appropriate engineering controls

Engineering controls are used to remove a hazard or place a barrier between the worker and the hazard. Well-designed engineering controls can be highly effective in protecting workers and will typically be independent of worker interactions to provide this high level of protection.
The basic types of engineering controls are:
Process controls which involve changing the way a job activity or process is done to reduce the risk.
Enclosure and/or isolation of emission source which keeps a selected hazard "physically" away from the worker and ventilation that strategically "adds" and "removes" air in the work environment. Ventilation can remove or dilute an air contaminant if designed properly. The design of a ventilation system must match the particular process and chemical or contaminant in use.
Employers may need to use multiple types of controls to prevent employee overexposure.

General exhaust is adequate under normal operating conditions. Local exhaust ventilation may be required in special circumstances. If risk of overexposure exists, wear approved respirator. Supplied-air type respirator may be required in special circumstances. Correct fit is essential to ensure adequate protection. Provide adequate ventilation in warehouses and enclosed storage areas. Air contaminants generated in the workplace possess varying "escape" velocities which, in turn, determine the "capture velocities" of fresh circulating air required to effectively remove the contaminant.

Type of Contaminant:	Air Speed:
solvent, vapours, degreasing etc., evaporating from tank (in still air).	0.25-0.5 m/s (50-100 f/min)
aerosols, fumes from pouring operations, intermittent container filling, low speed conveyer transfers, welding, spray drift, plating acid fumes, pickling (released at low velocity into zone of active generation)	0.5-1 m/s (100-200 f/min.)
direct spray, spray painting in shallow booths, drum filling, conveyer loading, crusher dusts, gas discharge (active generation into zone of rapid air motion)	1-2.5 m/s (200-500 f/min.)
grinding, abrasive blasting, tumbling, high speed wheel generated dusts (released at high initial velocity into zone of very high rapid air motion)	2.5-10 m/s (500-2000 f/min.)

Within each range the appropriate value depends on:

Lower end of the range	Upper end of the range
1: Room air currents minimal or favourable to capture	1: Disturbing room air currents
2: Contaminants of low toxicity or of nuisance value only.	2: Contaminants of high toxicity
3: Intermittent, low production.	3: High production, heavy use
4: Large hood or large air mass in motion	4: Small hood-local control only

Simple theory shows that air velocity falls rapidly with distance away from the opening of a simple extraction pipe. Velocity generally decreases with the square of distance from the extraction point (in simple cases). Therefore the air speed at the extraction point should be adjusted, accordingly, after reference to distance from the contaminating source. The air velocity at the extraction fan, for example, should be a minimum of 1-2 m/s (200-400 f/min) for extraction of solvents generated in a tank 2 meters distant from the extraction point. Other mechanical considerations, producing performance deficits within the extraction apparatus, make it essential that theoretical air velocities are multiplied by factors of 10 or more when extraction systems are installed or used.

8.2.2. Personal protection

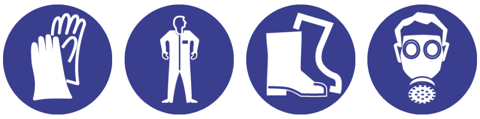

Eye and face protection

- Safety glasses with side shields
- Chemical goggles.
- Contact lenses may pose a special hazard; soft contact lenses may absorb and concentrate irritants. A written policy document, describing the wearing of lenses or restrictions on use, should be created for each workplace or task. This should include a review of lens absorption and adsorption for the class of chemicals in use and an account of injury experience. Medical and first-aid personnel should be trained in their removal and suitable equipment should be readily available. In the event of chemical exposure, begin eye irrigation immediately and remove contact lens as soon as practicable. Lens should be removed at the first signs of eye redness or irritation - lens should be removed in a clean environment only after workers have washed hands thoroughly. [CDC NIOSH Current Intelligence Bulletin 59], [AS/NZS 1336 or national equivalent]

Skin protection

See Hand protection below

Hands/feet protection

- Wear chemical protective gloves, e.g. PVC.
- Wear safety footwear or safety gumboots, e.g. Rubber
- Rubber Gloves

Body protection

See Other protection below

Other protection

No special equipment needed when handling small quantities.
OTHERWISE:
- Overalls.
- Barrier cream.
- Eyewash unit.

Respiratory protection

Continued...

Type K Filter of sufficient capacity. (AS/NZS 1716 & 1715, EN 143:2000 & 149:2001, ANSI Z88 or national equivalent)

Where the concentration of gas/particulates in the breathing zone, approaches or exceeds the "Exposure Standard" (or ES), respiratory protection is required.
Degree of protection varies with both face-piece and Class of filter; the nature of protection varies with Type of filter.

Required Minimum Protection Factor	Half-Face Respirator	Full-Face Respirator	Powered Air Respirator
up to 10 x ES	K-AUS	-	K-PAPR-AUS / Class 1
up to 50 x ES	-	K-AUS / Class 1	-
up to 100 x ES	-	K-2	K-PAPR-2 ^

^ - Full-face
A(All classes) = Organic vapours, B AUS or B1 = Acid gasses, B2 = Acid gas or hydrogen cyanide(HCN), B3 = Acid gas or hydrogen cyanide(HCN), E = Sulfur dioxide(SO2), G = Agricultural chemicals, K = Ammonia(NH3), Hg = Mercury, NO = Oxides of nitrogen, MB = Methyl bromide, AX = Low boiling point organic compounds(below 65 degC)

8.2.3. Environmental exposure controls

See section 12

SECTION 9 Physical and chemical properties

9.1. Information on basic physical and chemical properties

**Appearance**	Clear, colourless liquid with ammonia odour.

**Physical state**	Liquid	**Relative density (Water = 1)**	1.2
**Odour**	Not Available	**Partition coefficient n-octanol / water**	Not Available
**Odour threshold**	Not Available	**Auto-ignition temperature (°C)**	Not Available
**pH (as supplied)**	Not Available	**Decomposition temperature**	Not Available
**Melting point / freezing point (°C)**	Not Available	**Viscosity (cSt)**	Not Available
**Initial boiling point and boiling range (°C)**	Not Available	**Molecular weight (g/mol)**	Not Applicable
**Flash point (°C)**	Not Available	**Taste**	Not Available
**Evaporation rate**	Not Available	**Explosive properties**	Not Available
**Flammability**	Not Available	**Oxidising properties**	Not Available
**Upper Explosive Limit (%)**	Not Available	**Surface Tension (dyn/cm or mN/m)**	Not Available
**Lower Explosive Limit (%)**	Not Available	**Volatile Component (%vol)**	Not Available
**Vapour pressure (kPa)**	Not Available	**Gas group**	Not Available
**Solubility in water**	Not Available	**pH as a solution (1%)**	Not Available
**Vapour density (Air = 1)**	Not Available	**VOC g/L**	Not Available

9.2. Other information

Not Available

SECTION 10 Stability and reactivity

**10.1.Reactivity**	See section 7.2
**10.2. Chemical stability**	Product is considered stable and hazardous polymerisation will not occur.
**10.3. Possibility of hazardous reactions**	See section 7.2
**10.4. Conditions to avoid**	See section 7.2
**10.5. Incompatible materials**	See section 7.2
**10.6. Hazardous decomposition products**	See section 5.3

SECTION 11 Toxicological information

11.1. Information on toxicological effects

**Inhaled**	Limited evidence or practical experience suggests that the material may produce irritation of the respiratory system, in a significant number of individuals, following inhalation. In contrast to most organs, the lung is able to respond to a chemical insult by first removing or neutralising the irritant and then repairing the damage. The repair process, which initially evolved to protect mammalian lungs from foreign matter and antigens, may however, produce further lung damage resulting in the impairment of gas exchange, the primary function of the lungs. Respiratory tract irritation often results in an inflammatory response involving the recruitment and activation of many cell types, mainly derived from the vascular system.
**Ingestion**	The material can produce chemical burns within the oral cavity and gastrointestinal tract following ingestion.<br>Accidental ingestion of the material may be damaging to the health of the individual.
**Skin Contact**	The material can produce chemical burns following direct contact with the skin.<br>Open cuts, abraded or irritated skin should not be exposed to this material<br>Entry into the blood-stream through, for example, cuts, abrasions, puncture wounds or lesions, may produce systemic injury with harmful effects. Examine the skin prior to the use of the material and ensure that any external damage is suitably protected.

Continued...

**Eye**	The material can produce chemical burns to the eye following direct contact. Vapours or mists may be extremely irritating.
**Chronic**	Limited evidence suggests that repeated or long-term occupational exposure may produce cumulative health effects involving organs or biochemical systems.<br>Repeated or prolonged exposure to acids may result in the erosion of teeth, inflammatory and ulcerative changes in the mouth and necrosis (rarely) of the jaw. Bronchial irritation, with cough, and frequent attacks of bronchial pneumonia may ensue. Gastrointestinal disturbances may also occur. Chronic exposures may result in dermatitis and/or conjunctivitis.<br>The impact of inhaled acidic agents on the respiratory tract depends upon a number of interrelated factors. These include physicochemical characteristics, e.g., gas versus aerosol; particle size (small particles can penetrate deeper into the lung); water solubility (more soluble agents are more likely to be removed in the nose and mouth). Given the general lack of information on the particle size of aerosols involved in occupational exposures to acids, it is difficult to identify their principal deposition site within the respiratory tract. Acid mists containing particles with a diameter of up to a few micrometers will be deposited in both the upper and lower airways. They are irritating to mucous epithelia, they cause dental erosion, and they produce acute effects in the lungs (symptoms and changes in pulmonary function). AsthmatIcs appear to be at particular risk for pulmonary effects.

	TOXICITY	IRRITATION
**Riva Star**	Not Available	Not Available
**silver(I) fluoride**	Not Available	Not Available
**ammonia**	=750 mg/kg[2]	Eye (rabbit): 0.25 mg SEVERE
	20 mg/kg[2]	Eye (rabbit): 1 mg/30s SEVERE
	43 mg/kg[2]	
	Inhalation (rat) LC50: 1997.718 mg/l/4h[2]	
	Oral (rat) LD50: ~350-370 mg/kg[2]	
**water**	Oral (rat) LD50: >90000 mg/kg[2]	Not Available
Legend:	*1. Value obtained from Europe ECHA Registered Substances - Acute toxicity 2.* Value obtained from manufacturer's SDS. Unless otherwise specified data extracted from RTECS - Register of Toxic Effect of chemical Substances*	

**AMMONIA**	The material may produce severe irritation to the eye causing pronounced inflammation. Repeated or prolonged exposure to irritants may produce conjunctivitis.
**SILVER(I) FLUORIDE & AMMONIA**	Asthma-like symptoms may continue for months or even years after exposure to the material ceases. This may be due to a non-allergenic condition known as reactive airways dysfunction syndrome (RADS) which can occur following exposure to high levels of highly irritating compound. Key criteria for the diagnosis of RADS include the absence of preceding respiratory disease, in a non-atopic individual, with abrupt onset of persistent asthma-like symptoms within minutes to hours of a documented exposure to the irritant. A reversible airflow pattern, on spirometry, with the presence of moderate to severe bronchial hyperreactivity on methacholine challenge testing and the lack of minimal lymphocytic inflammation, without eosinophilia, have also been included in the criteria for diagnosis of RADS. RADS (or asthma) following an irritating inhalation is an infrequent disorder with rates related to the concentration of and duration of exposure to the irritating substance. Industrial bronchitis, on the other hand, is a disorder that occurs as result of exposure due to high concentrations of irritating substance (often particulate in nature) and is completely reversible after exposure ceases. The disorder is characterised by dyspnea, cough and mucus production.
**SILVER(I) FLUORIDE & AMMONIA & WATER**	No significant acute toxicological data identified in literature search.

**Acute Toxicity**	✔	**Carcinogenicity**	✘
**Skin Irritation/Corrosion**	✔	**Reproductivity**	✘
**Serious Eye Damage/Irritation**	✘	**STOT - Single Exposure**	✘
**Respiratory or Skin sensitisation**	✘	**STOT - Repeated Exposure**	✘
**Mutagenicity**	✘	**Aspiration Hazard**	✘

Legend: ✘ *– Data either not available or does not fill the criteria for classification*
✔ *– Data available to make classification*

SECTION 12 Ecological information

12.1. Toxicity

	Endpoint	Test Duration (hr)	Species	Value	Source
**Riva Star**	Not Available	Not Available	Not Available	Not Available	Not Available
**silver(I) fluoride**	Not Available	Not Available	Not Available	Not Available	Not Available

Continued...

**ammonia**	**Endpoint**	**Test Duration (hr)**	**Species**	**Value**	**Source**
	Not Available	Not Available	Not Available	Not Available	Not Available
**water**	**Endpoint**	**Test Duration (hr)**	**Species**	**Value**	**Source**
	Not Available	Not Available	Not Available	Not Available	Not Available
Legend:	*Extracted from 1. IUCLID Toxicity Data 2. Europe ECHA Registered Substances - Ecotoxicological Information - Aquatic Toxicity 3. EPIWIN Suite V3.12 (QSAR) - Aquatic Toxicity Data (Estimated) 4. US EPA, Ecotox database - Aquatic Toxicity Data 5. ECETOC Aquatic Hazard Assessment Data 6. NITE (Japan) - Bioconcentration Data 7. METI (Japan) - Bioconcentration Data 8. Vendor Data*				

Very toxic to aquatic organisms.
DO NOT discharge into sewer or waterways.

12.2. Persistence and degradability

Ingredient	Persistence: Water/Soil	Persistence: Air
water	LOW	LOW

12.3. Bioaccumulative potential

Ingredient	Bioaccumulation
water	LOW (LogKOW = -1.38)

12.4. Mobility in soil

Ingredient	Mobility
water	LOW (KOC = 14.3)

12.5.Results of PBT and vPvB assessment

	P	B	T
Relevant available data	Not Applicable	Not Applicable	Not Applicable
PBT Criteria fulfilled?	Not Applicable	Not Applicable	Not Applicable

12.6. Other adverse effects

No data available

SECTION 13 Disposal considerations

13.1. Waste treatment methods

**Product / Packaging disposal**	- **DO NOT** allow wash water from cleaning or process equipment to enter drains.<br>- It may be necessary to collect all wash water for treatment before disposal.<br>- In all cases disposal to sewer may be subject to local laws and regulations and these should be considered first.<br>- Where in doubt contact the responsible authority.<br>Consult State Land Waste Management Authority for disposal.<br>Bury residue in an authorised landfill.
**Waste treatment options**	Not Available
**Sewage disposal options**	Not Available

SECTION 14 Transport information

Labels Required

	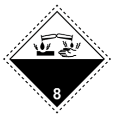
**Marine Pollutant**	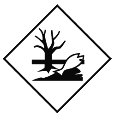
**HAZCHEM**	2X

Land transport (ADR)

14.1. **UN number**	2672
14.2. **UN proper shipping name**	AMMONIA SOLUTION, relative density between 0.880 and 0.957 at 15 °C in water, with more than 10% but not more than 35% ammonia

Continued...

14.3. **Transport hazard class(es)**	Class: 8<br>Subrisk: Not Applicable
14.4. **Packing group**	III
14.5. **Environmental hazard**	Environmentally hazardous
14.6. **Special precautions for user**	Hazard identification (Kemler): 80<br>Classification code: C5<br>Hazard Label: 8<br>Special provisions: 543<br>Limited quantity: 5 L<br>Tunnel Restriction Code: 3 (E)

Air transport (ICAO-IATA / DGR)

14.1. **UN number**	2672
14.2. **UN proper shipping name**	Ammonia solution relative density (specific gravity) between 0.880 and 0.957 at 15°C in water, with more than 10% but not more than 35% ammonia
14.3. **Transport hazard class(es)**	ICAO/IATA Class: 8<br>ICAO / IATA Subrisk: Not Applicable<br>ERG Code: 8L
14.4. **Packing group**	III
14.5. **Environmental hazard**	Environmentally hazardous
14.6. **Special precautions for user**	Special provisions: A64 A803<br>Cargo Only Packing Instructions: 856<br>Cargo Only Maximum Qty / Pack: 60 L<br>Passenger and Cargo Packing Instructions: 852<br>Passenger and Cargo Maximum Qty / Pack: 5 L<br>Passenger and Cargo Limited Quantity Packing Instructions: Y841<br>Passenger and Cargo Limited Maximum Qty / Pack: 1 L

Sea transport (IMDG-Code / GGVSee)

14.1. **UN number**	2672
14.2. **UN proper shipping name**	AMMONIA SOLUTION relative density between 0.880 and 0.957 at 15Â°C in water, with more than 10% but not more than 35% ammonia
14.3. **Transport hazard class(es)**	IMDG Class: 8<br>IMDG Subrisk: Not Applicable
14.4. **Packing group**	III
14.5. **Environmental hazard**	Marine Pollutant
14.6. **Special precautions for user**	EMS Number: F-A , S-B<br>Special provisions: Not Applicable<br>Limited Quantities: 5 L

Inland waterways transport (ADN)

14.1. **UN number**	2672
14.2. **UN proper shipping name**	AMMONIA SOLUTION, relative density between 0.880 and 0.957 at 15 °C in water, with more than 10% but not more than 35% ammonia
14.3. **Transport hazard class(es)**	8 | Not Applicable
14.4. **Packing group**	III
14.5. **Environmental hazard**	Environmentally hazardous
14.6. **Special precautions for user**	Classification code: C5<br>Special provisions: 543<br>Limited quantity: 5 L<br>Equipment required: PP, EP<br>Fire cones number: 0

14.7. Transport in bulk according to Annex II of MARPOL and the IBC code

Not Applicable

Continued...

If packed as Chemical kits the following classification may be considered if all ICAO/IATA transport requirements are met: Chemical Kit UN3316 - Class 9, SP A44 & A163.

SECTION 15 Regulatory information

15.1. Safety, health and environmental regulations / legislation specific for the substance or mixture

silver(I) fluoride is found on the following regulatory lists

EU Consolidated List of Indicative Occupational Exposure Limit Values (IOELVs)
Europe EC Inventory
European Union - European Inventory of Existing Commercial Chemical Substances (EINECS)
International Agency for Research on Cancer (IARC) - Agents Classified by the IARC Monographs
UK Workplace Exposure Limits (WELs)

ammonia is found on the following regulatory lists

Europe EC Inventory
European Union - European Inventory of Existing Commercial Chemical Substances (EINECS)
European Union (EU) Regulation (EC) No 1272/2008 on Classification, Labelling and Packaging of Substances and Mixtures - Annex VI

water is found on the following regulatory lists

Europe EC Inventory
European Union - European Inventory of Existing Commercial Chemical Substances (EINECS)

This safety data sheet is in compliance with the following EU legislation and its adaptations - as far as applicable - : Directives 98/24/EC, - 92/85/EEC, - 94/33/EC, - 2008/98/EC, - 2010/75/EU; Commission Regulation (EU) 2015/830; Regulation (EC) No 1272/2008 as updated through ATPs.

15.2. Chemical safety assessment

No Chemical Safety Assessment has been carried out for this substance/mixture by the supplier.

ECHA SUMMARY

Ingredient	CAS number	Index No	ECHA Dossier
silver(I) fluoride	7775-41-9	Not Available	Not Available

Harmonisation (C&L Inventory)	Hazard Class and Category Code(s)	Pictograms Signal Word Code(s)	Hazard Statement Code(s)
1	Skin Corr. 1B	GHS05; Dgr	H314

Harmonisation Code 1 = The most prevalent classification. Harmonisation Code 2 = The most severe classification.

Ingredient	CAS number	Index No	ECHA Dossier
ammonia	1336-21-6	007-001-01-2	01-2119982985-14-XXXX

Harmonisation (C&L Inventory)	Hazard Class and Category Code(s)	Pictograms Signal Word Code(s)	Hazard Statement Code(s)
1	Skin Corr. 1B; Aquatic Acute 1	GHS09; GHS05; Dgr	H314; H400

Harmonisation Code 1 = The most prevalent classification. Harmonisation Code 2 = The most severe classification.

Ingredient	CAS number	Index No	ECHA Dossier
water	7732-18-5	Not Available	Not Available

Harmonisation (C&L Inventory)	Hazard Class and Category Code(s)	Pictograms Signal Word Code(s)	Hazard Statement Code(s)
1	Not Classified	Not Available	Not Available

Harmonisation Code 1 = The most prevalent classification. Harmonisation Code 2 = The most severe classification.

National Inventory Status

National Inventory	Status
Australia - AIIC	Yes
Australia - Non-Industrial Use	No (silver(I) fluoride; ammonia; water)
Canada - DSL	No (silver(I) fluoride)
Canada - NDSL	No (ammonia; water)
China - IECSC	No (silver(I) fluoride)
Europe - EINEC / ELINCS / NLP	Yes
Japan - ENCS	Yes
Korea - KECI	Yes
New Zealand - NZIoC	Yes
Philippines - PICCS	No (silver(I) fluoride)
USA - TSCA	Yes
Taiwan - TCSI	Yes
Mexico - INSQ	No (silver(I) fluoride)
Vietnam - NCI	Yes
Russia - ARIPS	Yes

Continued...

National Inventory	Status
Legend:	*Yes = All CAS declared ingredients are on the inventory*<br>*No = One or more of the CAS listed ingredients are not on the inventory and are not exempt from listing(see specific ingredients in brackets)*

SECTION 16 Other information

**Revision Date**	03/09/2020
**Initial Date**	16/11/2015

Full text Risk and Hazard codes

**H311**	Toxic in contact with skin.
**H318**	Causes serious eye damage.
**H331**	Toxic if inhaled.
**H332**	Harmful if inhaled.

SDS Version Summary

Version	Issue Date	Sections Updated
6.1.1.1	01/11/2019	One-off system update. NOTE: This may or may not change the GHS classification
7.1.1.1	03/09/2020	Classification change due to full database hazard calculation/update.

Other information

Classification of the preparation and its individual components has drawn on official and authoritative sources as well as independent review by SDI Limited using available literature references.

The SDS is a Hazard Communication tool and should be used to assist in the Risk Assessment. Many factors determine whether the reported Hazards are Risks in the workplace or other settings. Risks may be determined by reference to Exposures Scenarios. Scale of use, frequency of use and current or available engineering controls must be considered.

For detailed advice on Personal Protective Equipment, refer to the following EU CEN Standards:
EN 166 Personal eye-protection
EN 340 Protective clothing
EN 374 Protective gloves against chemicals and micro-organisms
EN 13832 Footwear protecting against chemicals
EN 133 Respiratory protective devices

Definitions and abbreviations

PC－TWA: Permissible Concentration-Time Weighted Average
PC－STEL: Permissible Concentration-Short Term Exposure Limit
IARC: International Agency for Research on Cancer
ACGIH: American Conference of Governmental Industrial Hygienists
STEL: Short Term Exposure Limit
TEEL: Temporary Emergency Exposure Limit。
IDLH: Immediately Dangerous to Life or Health Concentrations
OSF: Odour Safety Factor
NOAEL :No Observed Adverse Effect Level
LOAEL: Lowest Observed Adverse Effect Level
TLV: Threshold Limit Value
LOD: Limit Of Detection
OTV: Odour Threshold Value
BCF: BioConcentration Factors
BEI: Biological Exposure Index

The information contained in the Safety Data Sheet is based on data considered to be accurate, however, no warranty is expressed or implied regarding the accuracy of the data or the results to be obtained from the use thereof.

Other information:
Prepared by: SDI Limited
3-15 Brunsdon Street, Bayswater Victoria, 3153, Australia
Phone Number: +61 3 8727 7111
Department issuing SDS: Research and Development
Contact: Technical Director
